# Acute Renal Failure in Patients with Severe Falciparum Malaria: Using the WHO 2006 and RIFLE Criteria

**DOI:** 10.1155/2013/841518

**Published:** 2013-01-29

**Authors:** Vipa Thanachartwet, Varunee Desakorn, Duangjai Sahassananda, Ko Ko Yazar Kyaw Win, Thanom Supaporn

**Affiliations:** ^1^Department of Clinical Tropical Medicine, Faculty of Tropical Medicine, Mahidol University, 420/6 Rajvithee Road, Ratchathevee, Bangkok 10400, Thailand; ^2^Phramongkutklao College of Medicine and Phramongkutklao Hospital, Bangkok 10400, Thailand

## Abstract

There are limited data on the application of the RIFLE criteria among patients with severe malaria. This retrospective study was conducted by reviewing 257 medical records of adult hospitalized patients with severe falciparum malaria at the Mae Sot General Hospital, Tak province in the northern part of Thailand. The aims of this study were to determine the incidence of acute renal failure (ARF) in patients with severe falciparum malaria and its association with RRT as well as in-hospital mortality. Using the WHO 2006 criteria, ARF was the second most common complication with incidence of 44.7% (115 patients). The requirement for RRT was 45.2% (52 patients) and the in-hospital mortality was 31.9% (36 patients). Using the RIFLE criteria, 73.9% (190 patients) had acute kidney injury (AKI). The requirement for RRT was 11.6% (5 patients) in patients with RIFLE-I and 44.9% (48 patients) in patients with RIFLE-F. The in-hospital mortality gradually increased with the severity of AKI. The requirement for RRT (*P* < 0.05) and the in-hospital mortality (*P* < 0.05) were significantly higher in ARF patients with severe falciparum malaria using both criteria. In conclusion, the RIFLE criteria could be used for diagnosing AKI and predicting outcomes in patients with severe malaria similar to the WHO 2006 criteria.

## 1. Introduction

Malaria is caused by protozoan parasites of the genus *Plasmodium,* namely, *P. falciparum*, *P. vivax*, *P. malariae*, *P. ovale,* and *P. knowlesi*. Patients with *P. falciparum* infection are prone to develop severe malaria in 30% of cases [1], which resulted in case fatality rate of 20% [2]. In Southeast Asia, acute renal failure (ARF) is one of the most common complications in adults with falciparum malaria [3, 4]. The incidence of ARF in patients with severe malaria varies widely ranging from 15% to 48% [5–11] which resulted in a high fatality rate of over 70% in untreated patients [12]. The availability of renal replacement therapy (RRT) and appropriate antimalarial chemotherapy has been shown to reduce case fatality rate as well as enhance the recovery of renal function [13, 14].

Recently, a few observational studies demonstrated that there was an increased risk for mortality with small increments in serum creatinine; this finding made the case for the adoption of more sensitive creatinine-based criteria for acute kidney injury (AKI) [15, 16]. The term ARF was replaced by AKI which was classified as the risk, injury, failure, loss, and end-stage renal failure criteria (RIFLE criteria) proposed by the Acute Dialysis Quality Initiative (ADQI) Group [17]. In addition, severity of AKI by the RIFLE criteria can be used for predicting both requirements for RRT and mortality rates particularly in critically ill patients [17]. However, data on diagnosing AKI using the RIFLE criteria among patients with severe malaria are limited due to the commonly used WHO criteria in this field [18].

This study aimed to determine the incidence of ARF by the WHO 2006 criteria and AKI by the RIFLE criteria as well as their association with requirement for RRT and in-hospital mortality.

## 2. Patients and Methods

### 2.1. Study Design

 This study was approved by the Ethics Committee of the Faculty of Tropical Medicine, Mahidol University, Bangkok, Thailand. A retrospective study was conducted at the Mae Sot General Hospital, Tak province, Thailand. Medical records of hospitalized patients with severe falciparum malaria classified according to WHO 2006 criteria [18] between 2004 and 2008 were reviewed. Inclusion criteria were (1) aged 15 years or above, (2) confirmed diagnosis of falciparum malaria by peripheral blood smear microscopy, and (3) had clinical and laboratory features of severe malaria as defined by the WHO 2006 criteria [18]. Exclusion criteria were (1) patients with previous history of chronic kidney disease or (2) patients with mixed infection.

 Patients' data comprising demographic, clinical features, laboratory data, and outcomes including death and requirement for RRT were reviewed and extracted from medical records and discharge summaries according to the eligible criteria. These data were then transferred into a predefined case record form for further analysis. 

### 2.2. Case Definitions

In our study, the WHO 2006 criteria [18] for severe malaria were used with slight modification due to the availability of data. These included (1) impaired consciousness defined as Glasgow coma scale (GCS) score ≤10, (2) multiple convulsions defined as patients who developed convulsion ≥2 times within 24 hours, (3) shock defined as a systolic blood pressure ≤80 mmHg or required inotropic drugs, (4) pulmonary edema or acute respiratory distress syndrome (ARDS) assessed by clinical and abnormal chest roentgenography specific for these syndromes, (5) hyperbilirubinemia defined as total bilirubin ≥3 mg/dL, (6) hypoglycemia defined as blood sugar <40 mg/dL, (7) severe anemia defined as hematocrit <15% or hemoglobin <5 g/dL, (8) spontaneous bleeding, (9) evidence of disseminated intravascular coagulation, (10) severe metabolic acidosis defined as arterial blood pH <7.35 or bicarbonate level <15 mmol/L, and (11) hemoglobinuria defined as dark colored urine or heme positive with <2 red blood cells per high-power field in urine.

Patients with ARF were classified according to the WHO 2006 criteria defined as serum creatinine >3 mg/dL and adequate volume status [18]. Patients with AKI were classified according to the RIFLE criteria using decrement in the estimated glomerular filtration rate (GFR), but not serum creatinine increment and urine output in our study. Patients with AKI were classified as no AKI (decrease of estimated GFR ≤25%), RIFLE-R (decrease of estimated GFR >25–50%), RIFLE-I (decrease of estimated GFR >50–75%) and RIFLE-F (decrease of estimated GFR >75%), using the worst RIFLE category during hospitalization [17]. The categories of RIFLE including loss (RIFLE-L) and end-stage kidney disease (RIFLE-E) were not evaluated. Estimated GFR was calculated using the Chronic Kidney Disease Epidemiology Collaboration (CKD-EPI) equation as recommended by the ADQI group [19]. 

### 2.3. Sample Size Calculation

The incidence of ARF in adults with severe malaria was approximately 30% to 50% by the reports from South and Southeast Asia [8, 9, 11]. Data from the hospital registry of the Mae Sot General Hospital showed that 25% of adult patients with severe malaria had ARF; thus, we estimated the incidence of ARF in our study to be 25% with the desired statistical power of 80% and the type I error (alpha = 0.05) to detect a 10% difference in incidence of AKI between the WHO 2006 criteria and the RIFLE criteria. The required sample size of at least 231 medical records of hospitalized patients with severe falciparum malaria was needed in our study.

### 2.4. Statistical Analysis

 The demographic, clinical, and laboratory data collected in this study were analyzed using the Statistical Package for the Social Sciences version 18.0 (SPSS, Chicago, IL, USA). Quantitative data were tested for normality using the Kolmogorov-Smirnov test and summarized as median (interquartile, IQR) for nonnormally distributed data. Qualitative data were summarized as frequency (percentage) and then analysed by Chi square test or Fisher's exact test as appropriate. The Chi square for linear trend was used to determine a linear trend of in-hospital mortality in relation to severity of AKI. Univariate analysis was performed to determine the possible risk factors for in-hospital mortality among severe falciparum malaria patients with AKI. Any variable with *P* ≤ 0.2 in the univariate logistic regression analysis was included in the stepwise multivariate logistic regression analysis using a backward selection method for determining the independent associated factors for in-hospital mortality among severe falciparum malaria patients with AKI. All tests of significance were 2 sided, with a *P* < 0.05 indicating statistical significance.

## 3. Results

A total of 373 medical records of hospitalized patients with severe falciparum malaria were reviewed. Of these, 257 medical records fulfilled the eligible criteria and the rest were excluded as 86 patients had insufficient data and 30 patients had mixed infection. The median (IQR) age of patients with severe falciparum malaria was 31.0 (22.0–40.0) years and the majority of the patients were males (191/257, 74.3%). The common clinical presentations on admission were fever (245/247, 99.2%) with the median (IQR) temperature of 38.7 (37.7–39.5)°C and rigors (102/109, 93.6%). 

 Regarding laboratory parameters on admission, hematological findings were hemoglobin (median (IQR), 10.6 (8.3–12.9) g/dL), white blood cell count (8.3 (5.4–12.3) × 10^9^/L), and platelet count (32.0 (17.0–64.0) × 10^9^/L). Blood chemistries showed blood sugar (median (IQR), 123 (98–146) mg/dL), blood urea nitrogen (44.0 (22.0–72.8) mg/dL), creatinine (1.8 (1.2–4.1) mg/dL), total bilirubin (5.0 (2.2–11.1) mg/dL), direct bilirubin (1.5 (0.7–3.8) mg/dL), aspartate aminotransferase (124 (59–232) U/L), and alanine aminotransferase (60 (32–112) U/L).

According to the WHO 2006 criteria, most of the patients had complications of hyperbilirubinemia (139, 54.1%) followed by ARF (115, 44.7%), impaired consciousness (110, 42.8%), severe metabolic acidosis (109, 42.4%), shock (86, 33.5%), multiple convulsions (30, 11.7%), pulmonary edema or ARDS (23, 8.9%), severe anemia (16, 6.2%), hypoglycemia (13, 5.1%) and spontaneous bleeding (7, 2.7%). The incidence of ARF using the WHO 2006 and RIFLE criteria are shown in Table 1. For the WHO 2006 criteria, ARF occurred in 115 patients (44.7%) during admission. Using the RIFLE criteria, AKI was observed in 190 patients (73.9%) including RIFLE-R (40/257, 15.6%), RIFLE-I (43/257, 16.7%), and RIFLE-F (107/257, 41.6%).

### 3.1. RRT Requirement

 Regarding the WHO 2006 criteria, 53 of 257 (20.6%) patients underwent dialysis (31 patients by hemodialysis and 22 patients by peritoneal dialysis) with median (IQR) duration for dialysis of 4.5 (2.0–10.0) days. Of 53 patients, 52 patients were classified as a group with ARF and only one patient as a group with no ARF. The requirement for RRT was significantly higher in the group with ARF compared to that with no ARF (*P* < 0.001). Using the RIFLE criteria, none of the patients with no AKI and RIFLE-R received RRT whereas 53 patients (27.9%) including RIFLE-I (5/43, 11.6%) and RIFLE-F (48/107, 44.9%) received RRT. The requirement for RRT in a group with any RIFLE criteria was significantly higher than that with no AKI (*P* < 0.001) (Table 2). 

The most common indication for RRT in patients with RIFLE-I was severe metabolic acidosis (4, 80.0%) followed by pulmonary edema (2, 40.0%) and severe hyperkalemia (2, 40.0%). Among patients with RIFLE-F, the most common indication for RRT was severe metabolic acidosis (25, 52.1%) followed by pulmonary edema (9, 18.6%), severe hyperkalemia (6, 12.5%), fluid management for nutrition support (2, 4.2%), and uremia (1, 2.1%).

### 3.2. In-Hospital Mortality

Using the WHO 2006 criteria, in-hospital mortality of patients with ARF was significantly higher than that with no ARF (36/113 (31.9%) versus 19/136 (14.0%); *P* = 0.001). When patients were classified by the RIFLE criteria, in-hospital mortality was significantly higher in the group of patients with any RIFLE criteria compared to that with no AKI (49/187 (26.2%) versus 6/62 (9.7%); *P* = 0.002). The results showed that in-hospital mortality increased with severity of AKI including RIFLE-R (4/39, 10.3%), RIFLE-I (13/43, 30.2%), and RIFLE-F (32/105, 30.5%) (Table 2), and there was strong evidence of a linear trend in-hospital mortality in relation to severity of AKI (Chi square for linear trend = 12.693, *P* < 0.001). Patients with RIFLE-R, RIFLE-I, and RIFLE-F were 1.07, 4.04, and 4.09 times at risk for in-hospital mortality as compared to those with no AKI, respectively.

### 3.3. Univariate and Multivariate Logistic Regression Analysis of Risk Factors for In-Hospital Mortality among Severe Falciparum Malaria Patients with AKI

 The clinical, laboratory parameters and the type of RRT were subsequently analyzed for independent factors associated with in-hospital mortality among severe falciparum malaria patients with AKI (Table 3). Univariate analysis showed that GCS ≤ 10 (odds ratio (95% confidence interval); OR (95% CI), 8.477 (3.852–18.654), *P* < 0.001); number of WHO criteria ≥4 (22.500 (7.625–66.396), *P* < 0.001), dopamine infusion (17.185 (5.945–49.679), *P* < 0.001), adrenaline infusion (130.500 (34.422–494.744), *P* < 0.001), need for mechanical ventilator (37.266 (12.450–111.546), *P* < 0.001), white blood cell count >12.0 × 10^9^/L (5.286 (2.602–10.736), *P* < 0.001), serum potassium >5.5 mmol/L (10.427 (2.731–39.808), *P* = 0.001), bicarbonate level <15 mmol/L (6.195 (2.509–15.295), *P* < 0.001), blood sugar <60 mg/dL (12.000 (1.379–104.410), *P* = 0.024), aspartate aminotransferase >500 U/L (7.429 (2.646–20.858), *P* < 0.001), alanine aminotransferase >500 U/L (13.875 (1.497–128.561), *P* = 0.021) and albumin <2.5 g/dL (2.597 (1.205–5.597), *P* = 0.015) were significantly associated with in-hospital mortality at *P* ≤ 0.2. These parameters were further analyzed by stepwise multivariate logistic regression analysis using backward selection method. The parameters including dopamine infusion (OR (95% CI), 7.172 (1.827–28.145), *P* = 0.005), adrenaline infusion (14.502 (2.874–73.166), *P* = 0.001), need for mechanical ventilator (10.806 (2.569–45.459), *P* = 0.001), and white blood cell count >12.0 ×10^9^/L (3.982 (1.146–13.836), *P* = 0.030) were independently associated with in-hospital mortality.

## 4. Discussion

*P. falciparum* infection is the most common cause of ARF in patients with severe malaria [3, 4]. It appears that several factors contribute to ARF in falciparum malaria which includes parasitized erythrocytes inducing microvascular obstruction and/or causing hemolysis [4]. Apart from parasites, glycosyl-phosphatidyl-inositol which is a receptor on monocytes covalently bound to the surface antigens of falciparum malaria parasites. The monocytes are then stimulated to release the tumor necrosis factor, which in turn enhances synthesis of various cytokine cascades and mediators. These mediators also cause changes in blood volume status, vasodilatation, and increase vascular permeability resulting in hypovolemia which contributes to ischemic renal failure [3].

We conducted a retrospective study by reviewing 257 medical records of patients with severe falciparum malaria at the Mae Sot General Hospital, Tak province, Thailand, in order to determine the incidence of ARF using the WHO 2006 criteria and AKI using the RIFLE criteria as well as their association with RRT requirement and in-hospital mortality. Serum creatinine was used to calculate estimated GFR as serum creatinine criteria seemed to show a worse RIFLE category and provided a better predictor for mortality rate than urine output criteria [20]. However, calculation of estimated GFR has several limitations particularly in AKI patients with nonsteady state of serum creatinine. The lack of steady state in serum creatinine in AKI patients might lead to an overestimation of GFR in patients with rising serum creatinine and underestimation of GFR in patients with declining GFR.

Regarding the WHO 2006 criteria, the incidence of ARF in our study (44.7%) was similar to the reports in previous studies (30–50%) [8, 9, 11]. Overall in-hospital mortality of patients with severe falciparum malaria was 22.1% and as high as 31.9% in the group of patients with ARF in our study. This figure showed significantly higher in-hospital mortality in the group of patients with ARF compared to that with no ARF (14%). The cause of death was multiorgan failure, which included shock, cerebral malaria, and metabolic acidosis in most cases. These findings were similar to reports in other referral centers showing that ARF occurred in approximately 25–50%, and the numbers of organ failures were associated with case fatality rate [7, 8, 11, 14]. When RRT requirement was observed, indications for initiation of RRT in patients with severe malaria were similar to the timing for initiation of RRT in critically ill patients and dependent on treating nephrologists. However, the indication for initiation of RRT included severe metabolic acidosis, pulmonary edema, and severe hyperkalemia in our hospital. Our study showed that 45.2% of severe falciparum malaria patients with ARF required RRT. However, the proportion for the requirement of RRT in our study was lower than those reports in other studies (60–80%) [7, 8, 11, 14].

Recently, the ADQI group established the RIFLE criteria for diagnosing AKI. These criteria have been shown in hospitalized patients to be quite sensitive in predicting the case fatality rate [15–17, 21]. There were several reports showing the ability of RIFLE criteria in predicting hospital mortality of critically ill patients [22–25]. These criteria have been widely used for diagnosing AKI in western countries, but they are rarely used in tropical areas, of which infection is one of the most common causes of AKI. Infectious diseases in these tropical countries including malaria, leptospirosis, scrub typhus, and salmonellosis are commonly found in association with AKI [26]. 

There has been only one report from India showing that AKI diagnosed using the RIFLE criteria was associated with the requirement for RRT and case fatality rate in patients with tropical acute febrile illnesses such as scrub typhus, falciparum malaria, enteric fever, dengue, and leptospirosis [27]. However, these RIFLE criteria have never been evaluated among a cohort of patients with severe malaria. In our study, AKI was evaluated using the RIFLE criteria in a large group of patients with severe malaria. We demonstrated that the requirement for RRT and in-hospital mortality increased significantly with severity of AKI. Severe malaria patients with RIFLE-I or RIFLE-F were 4 times at risk for in-hospital mortality compared to those with no AKI. Risk factors for in-hospital mortality among severe falciparum malaria patients with AKI were dopamine (OR = 7.172, 95% CI = 1.827–28.145) or adrenaline infusion (OR = 14.502, 95% CI = 2.874–73.166), need for mechanical ventilator (OR = 10.806, 95% CI = 2.569–45.459), and white blood cell count >12.0 ×10^9^/L (OR = 3.982, 95% CI = 1.146–13.836) in our study. Previous studies showed that in-hospital mortality among patients with severe malaria was higher in those with multi-organ failure [28]. Furthermore, leucocytosis was associated with mortality among patients with falciparum malaria but not associated with bacteremia [29]. Early detection of AKI may help in the proper management of cases resulting in better outcomes. Other conditions such as cerebral malaria, disseminated intravascular coagulation, and metabolic acidosis may contribute to poor outcomes in patients with severe falciparum malaria [5, 8].

In our study, there were several limitations due to the study design which was retrospective in nature and some baseline data of the patients were missing. Therefore, further prospective evaluations are needed to properly standardize the RIFLE criteria for diagnosing AKI in malaria patients.

In conclusion, RIFLE criteria could be used in diagnosing AKI and predicting both requirements for RRT and in-hospital mortality in patients with severe falciparum malaria similar to WHO 2006 criteria. Early diagnosis and early management of AKI may help to improve the outcomes of severe malaria patients in future. 

## Figures and Tables

**Table 1:** Incidence of acute renal failure classified by the WHO 2006 and RIFLE* criteria.

Criteria	No. (%)
(a) WHO criteria (*n* = 257)	
No ARF^†^	142 (55.3)
ARF^†^	115 (44.7)
(b) RIFLE* criteria (*n* = 257)	
No AKI^‡^	67 (26.1)
Any RIFLE*	190 (73.9)
Risk	40 (15.6)
Injury	43 (16.7)
Failure	107 (41.6)

*RIFLE: risk, injury, failure, loss, and end-stage renal failure. ^†^ARF: acute renal failure. ^‡^AKI: acute kidney injury.

**Table 2:** Severe falciparum malaria patients with acute renal failure using the WHO 2006 and RIFLE* criteria in relation to RRT^†^ requirement and in-hospital mortality.

Criteria	RRT^†^ (*n* = 257) no. (%)			Death (*n* = 249) no. (%)		
	No	Yes	*P* value	No	Yes	*P* value
WHO 2006						
No ARF^‡^	141 (99.3)	1 (0.7)		117 (86.0)	19 (14.0)	
ARF^‡^	63 (54.8)	52 (45.2)	<0.001	77 (68.1)	36 (31.9)	0.001
RIFLE*						
No AKI^#^	67 (100.0)	0 (0.0)		56 (90.3)	6 (9.7)	
Any RIFLE*	137 (72.1)	53 (27.9)	<0.001	138 (73.8)	49 (26.2)	0.002
Risk	40 (100.0)	0 (0.0)		35 (89.7)	4 (10.3)	
Injury	38 (88.4)	5 (11.6)		30 (69.8)	13 (30.2)	
Failure	59 (55.1)	48 (44.9)		73 (69.5)	32 (30.5)	

*RIFLE: risk, injury, failure, loss, and end-stage renal failure. ^†^RRT: renal replacement therapy. ^‡^ARF: acute renal failure. ^#^AKI: acute kidney injury.

**Table 3:** Univariate and multivariate logistic regression analysis of risk factors for in-hospital mortality among severe falciparum malaria patients with acute kidney injury.

Parameters	Univariate analysis		Multivariate analysis*	
	OR^†^ (95% CI^‡^)	*P* value	OR^†^ (95% CI^‡^)	*P* value
Glasgow coma scale ≤10	8.477 (3.852–18.654)	<0.001		
Number of WHO criteria ≥4	22.500 (7.625–66.396)	<0.001		
Inotropic drug				
No	1.000		1.000	
Dopamine	17.185 (5.945–49.679)	<0.001	7.172 (1.827–28.145)	0.005
Adrenaline	130.500 (34.422–494.744)	<0.001	14.502 (2.874–73.166)	0.001
Mechanical ventilator	37.266 (12.450–111.546)	<0.001	10.806 (2.569–45.459)	0.001
WBC^#^ >12.0 × 10^9^/L	5.286 (2.602–10.736)	<0.001	3.982 (1.146–13.836)	0.030
Potassium >5.5 mmol/L	10.427 (2.731–39.808)	0.001		
Bicarbonate <15 mmol/L	6.195 (2.509–15.295)	<0.001		
Blood sugar <60 mg/dL	12.000 (1.379–104.410)	0.024		
AST^€^ >500 U/L	7.429 (2.646–20.858)	<0.001		
ALT^*||*^ >500 U/L	13.875 (1.497–128.561)	0.021		
Albumin ≤2.5 mg/dL	2.597 (1.205–5.597)	0.015		

*Unaffected factors including the Glasgow coma scale, total WHO criteria, potassium, bicarbonate, blood sugar, ALT^*||*^, and albumin.^†^OR: odds ratio. ^‡^CI: confidence intervals. ^#^WBC: white blood cell count. ^€^AST: aspartate aminotransferase. ^*||*^ALT: alanine aminotransferase.
